# Purposes and characteristics of virtual reality technologies for the elderly in the community: a scoping review *

**DOI:** 10.1590/1518-8345.7419.4389

**Published:** 2024-11-22

**Authors:** Jamylle Lucas Diniz, Natalia Maria Cavalcante Oliveira, Janaina Fonseca Victor Coutinho, Marília Braga Marques, Carolina Bravo Pillon, Ítalo Linhares de Araújo

**Affiliations:** ^1^ Universidade Federal do Ceará, Departamento de Enfermagem, Fortaleza, CE, Brazil.; ^2^ Scholarship holder at the Coordenação de Aperfeiçoamento de Pessoal de Nível Superior (CAPES), Brazil.; ^3^ Universidade Federal de Pelotas, Centro de Artes, Pelotas, RS, Brazil.; ^4^ Universidade Federal do Ceará, Campus Itapajé, Itapajé, CE, Brazil.

**Keywords:** Technology, Virtual Reality, Elderly, Home, Postural Balance, Rehabilitation

## Abstract

to map the characteristics and purposes of Virtual Reality (VR) technologies for the elderly in the community.

scoping review, according to JBI recommendations and described according to the Preferred Reporting Items for Systematic Reviews and Meta-Analyses Extension for Scoping Reviews. Ten databases and four platforms referring to gray literature were included. Studies were selected after duplicates were removed and individual and peer reviews were carried out. Similarity analysis was used to identify competition between words and their results.

20 studies on VR for the elderly were mapped, the majority of which used non-immersive devices. The main purpose of using VR with the elderly is to improve and/or rehabilitate functions that decline with aging, either physiologically or as a result of illness or injury. VR devices are a potential tool for the prevention of falls and cognitive decline and favor the performance of instrumental activities of daily living. Similarity analysis resulted in the generation of a maximum tree, which identified the interrelationship between the terms “virtual reality” and “elderly” as the central and intermediate elements, respectively.

it is recommended that further studies be carried out in other environments, which could allow for a wider use of VR by health professionals, especially nurses, in the care provided to the elderly.

## Highlights

**(1)** Improved cognitive function, balance and mobility in elderly people.

**(2)** Promotes the execution of instrumental activities of daily living.

**(3)** A greater number of non-immersive devices were identified.

**(4)** Virtual Reality devices are recognized as useful, easy to use and a pleasant experience.

**(5)** It is recommended that new studies be developed in other health environments.

## Introduction

 Innovative and complementary forms of healthcare have been developed through technological solutions aimed at helping, maintaining, and rehabilitating the elderly, including the Internet of Health Things (IoHT) ^(^ 1 ^)^ , wearable devices ^(^ 2 ^)^ , Augmented Reality (AR), Virtual Reality (VR) and Mixed Reality (MR) ^(^ 3 ^-^ 6 ^)^ . 

 As far as virtual technologies are concerned, we are interested in VR devices that make it possible to diversify experiences depending on the individual and the environment. Intervention studies using VR have already been used in the field of education during the COVID-19 pandemic and in healthcare for the treatment of chronic pain, rehabilitation after a stroke, and the control of depression symptoms ^(^ 7 ^-^ 10 ^)^ . 

 In 2020, the global VR market generated approximately US$ 10.85 billion and by 2028 it could reach US$ 52.03 billion, an increase of 21.9%. North America stands out in terms of development and commercialization, with the health and education sectors placing the greatest demand on the development of VR devices ^(^ 11 ^)^ . 

 VR can be used by different age groups, but there is a difference in demand: young people use it for entertainment ^(^ 12 ^)^ , while the elderly see it as a care tool for health professionals, especially to improve physical (posture, balance, gait, range of movement, expenditure) cognitive (executive function, attention, memory, symptoms of depression, anxiety, mood) ^(^ 13 ^)^ and social aspects. 

 VR devices show the potential for positive changes in the well-being and quality of life of the elderly ^(^ 14 ^-^ 15 ^)^ . However, they are not without risk and can cause discomfort during use, such as nausea, dizziness, vertigo, and headaches. These symptoms are known as “cyber-sickness”, which may be related to the type of device, exposure time, predisposition, and non-adaptation ^(^ 16 ^)^ . 

 VR devices for the elderly have different characteristics and purposes. Knowing these will allow health professionals, especially nurses, to evaluate new possibilities for interventions and improve the care offered. Thus, by investigating and mapping VR devices in the literature, the possibilities of use, attributes, and possible discomforts can be assessed. In addition to helping to choose the best type, knowing which ones are the most accepted and suitable, it also provides the information to create standardized checklists, with their own interface and functionality specifications for the development of VR devices aimed specifically at the elderly population ^(^ 12 ^)^ . 

This study aimed to map out the characteristics and purposes of virtual reality technologies for the elderly in the community.

## Method

### Study design

 This is a scoping review (SR) that adopted the recommendations of the Joanna Briggs Institute (JBI) ^(^ 17 ^)^ , registered in the Open Science Framework with DOI 10.17605/OSF.IO/YGZ9Q. This was followed by the Reporting Items for Systematic Reviews and Meta-analyses Extension for Scoping Reviews (PRISMA-ScR) ^(^ 18 ^)^ . 

### Scenario in which the data was collected

The search was carried out in the following databases: Medical Literature Analysis and Retrieval System Online/National Library of Medicine (MEDLINE/PubMed), Cumulative Index to Nursing and Allied Health Literature (CINAHL), Scopus, Embase, JBI Evidence Synthesis, Epistemonikos, Compendex, PsycINFO, Cochrane Library and Web of Science. For gray literature, the sources used were: Google Scholar (first ten pages); Continental Europe - System for Information on Grey Literature in Europe (OpenGrey); ProQuest Global Dissertations and Theses (first ten pages), and ClinicalTrials.

### Timeframe

The research was carried out between April and May 2023.

### Population

The mnemonic Population; Concept and Context (PCC) was used, Population (P) - elderly people characterized by individuals aged 60 or over. Concept (C), use of VR technologies and Context (Ct) - community (studies conducted in community environments, or covering the home, not involving elderly people under institutional care). The research question was: What are the characteristics and purpose of virtual reality technologies developed for the elderly in the community?

### Selection criteria

 The eligibility criteria were organized using the PCC strategy ^(^ 17 ^)^ . Studies investigating the use of VR technologies by elderly people in the community were included, with no restrictions on the year of publication or language. We excluded review studies, letters, congress proceedings, study protocols, and research conducted in hospitals and long-term care facilities, as they covered elderly people in institutional care and duplicate articles. 

### Sample definition

**Figure 1 -:**
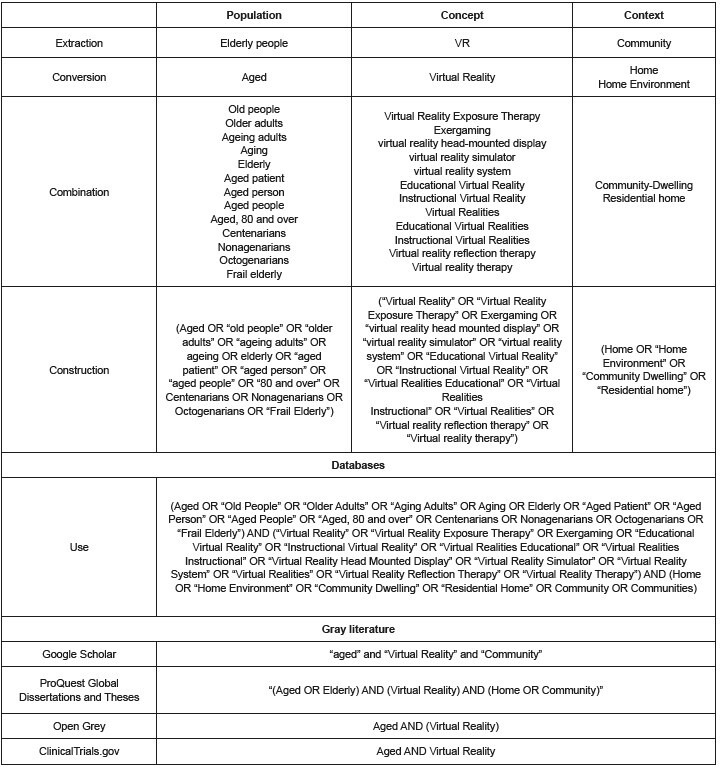
Elaboration of the search strategy in databases and gray literature. Fortaleza, CE, Brazil, 2023

 Controlled descriptors from the Medical Subject Headings (MeSH), Medical Subject Heading (MeSH), and Emtree vocabularies and keywords were used. To do this, a simple search was carried out on MEDLINE/PubMed and Scopus using the PCC descriptors, to analyze the words contained in the title and abstract, as well as indexing the terms used to describe the articles. This phase was carried out by the researcher with the help of a librarian from the Federal University of Ceará. Subsequently, search commands were created with the words retrieved together with the descriptors, including their synonyms and keywords according to their relevance to the study. The cross-referencing was mediated by the Boolean operators AND and OR. The search strategy was thus structured into five stages: extraction, conversion, combination, construction, and use ^(^ 19 ^)^ , as shown in Figure 1 . 

### Data collection

The search, evaluation, selection, characterization, and analysis procedures were carried out by two researchers, who were paired up and held consensus meetings to deal with the decision-making process and the inclusion of articles. The Rayyan application, developed by the Qatar Computing Research Institute (QCRI), was used to help with filing, organization, and selection.

### Study variables

The variables used were: authorship, year of publication, language and country of origin, type of study, professionals involved, characteristics of the technologies, purpose of VR, side effects resulting from the use of VR, and the main conclusions.

### Instruments used to collect information

The mapping of information was based on the JBI instrument to characterize the productions.

### Data processing and analysis

 The results were subjected to descriptive analysis, using the software *Interface de R pour les Analyses Multidimensionnelle de Textes et de Questionnaires* (IRAMuTeQ), 0.7 Alpha 2. Similarity analysis was carried out, characterized by the fact that it enables the identification of co-occurrences between words and their result, providing indications of the connection between them through graphs ^(^ 20 ^)^ . 

### Ethical aspects

This is a study using secondary data, so there was no need for the Research Ethics Committee (REC).

## Results

**Figure 2 -:**
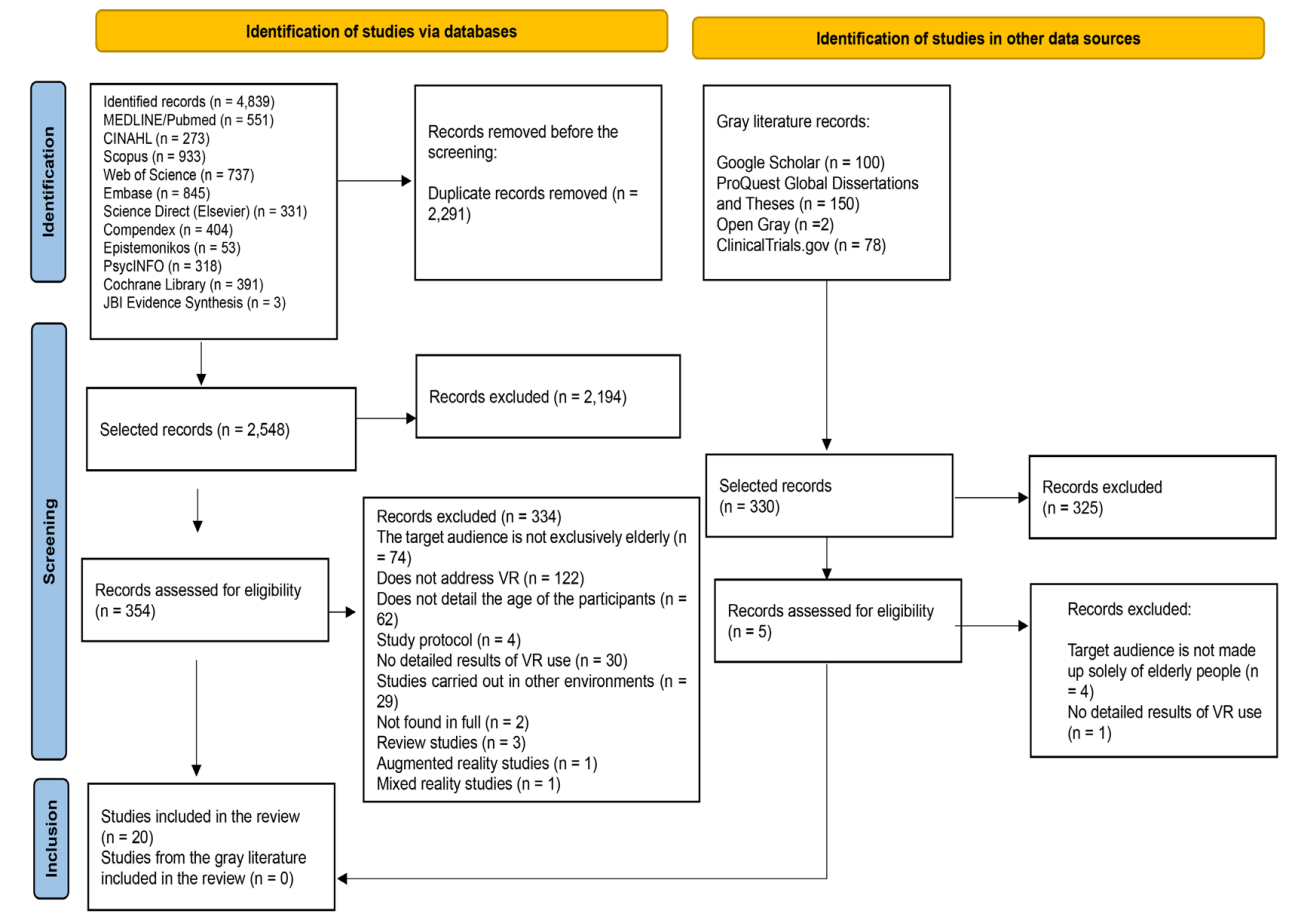
Study selection flowchart. Fortaleza, CE, Brazil, 2023

 A total of 4,839 articles were identified in the databases and 330 in the grey literature, totaling 5,169, of which 2,291 were excluded due to duplicate publications, leaving 2,878 for reading the title and abstract. After the first analysis, reading the titles and abstracts screened, 2,848 were excluded and 360 were selected to be read in full. Of these, 340 were excluded because they did not answer the research question. The final sample consisted of 20 articles ^(^ 21 ^-^ 40 ^)^ , as shown in Figure 2 . 

 As for the country of origin of the studies, six were carried out in South Korea ^(^ 21 ^-^ 23 ^,^ 26 ^,^ 30 ^,^ 38 ^)^ , three in Greece ^(^ 27 ^,^ 31 ^,^ 36 ^)^ , two in the USA ^(^ 37 ^,^ 39 ^)^ and Taiwan ^(^ 29 ^,^ 34 ^)^ , and one each in Singapore ^(^ 24 ^)^ , Iran ^(^ 25 ^)^ , Poland ^(^ 32 ^)^ , Portugal ^(^ 33 ^)^ , Canada ^(^ 35 ^)^ , Australia ^(^ 40 ^)^ , respectively, and one multicenter study ^(^ 28 ^)^ involving Belgium, Israel, Italy, the Netherlands, and the United Kingdom. As for the year of publication, four articles were published in 2020 ^(^ 22 ^,^ 33 ^-^ 35 ^)^ and 2017 ^(^ 30 ^-^ 31 ^,^ 37 ^,^ 40 ^)^ , and three in 2019 ^(^ 23 ^,^ 29 ^,^ 39 ^)^ . Followed by two in 2021 ^(^ 21 ^,^ 25 ^)^ , in 2016 ^(^ 24 ^,^ 28 ^)^ and one in 2022 ^(^ 32 ^)^ , 2018 ^(^ 36 ^)^ , 2015 ^(^ 26 ^)^ , 2013 ^(^ 38 ^)^ and 2007 ^(^ 27 ^)^ , respectively. 

**Figure 3 -:**
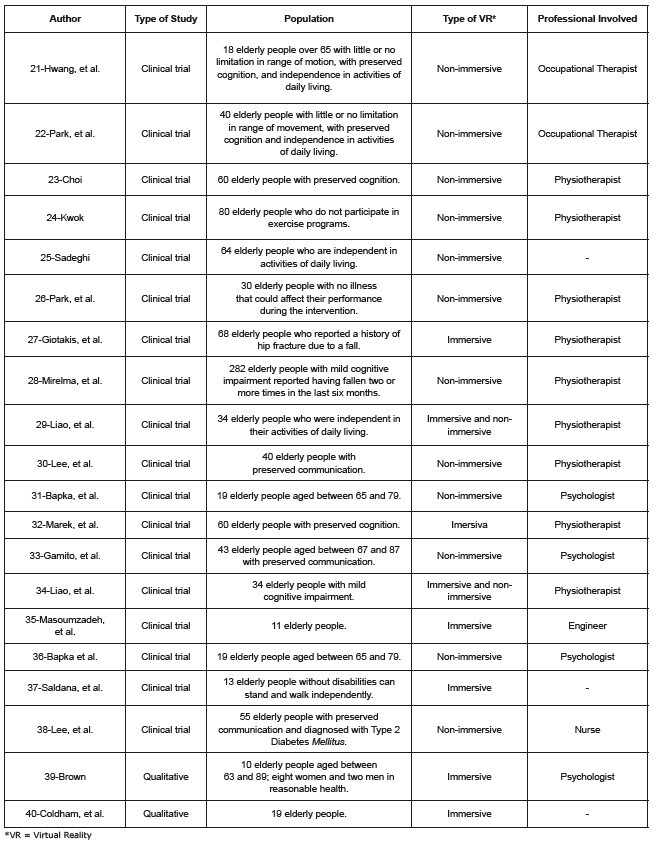
Description of the selected studies (20) according to the author, type of study, population, type of VR*, and professional involved. Fortaleza, CE, Brazil, 2023

 The instruments used in the studies were the Mini-Mental State Examination (MMSE); the Manual Muscle Test of the upper and lower extremities; the Montreal Cognitive Assessment (MOCa); the Modified Falls Effectiveness Scale (MFES); the Dementia Clinical Assessment Scale and the Berg Balance Scale. The description of the studies according to population, inclusion criteria, and professionals involved are condensed in Figure 3 . 

**Figure 4 -:**
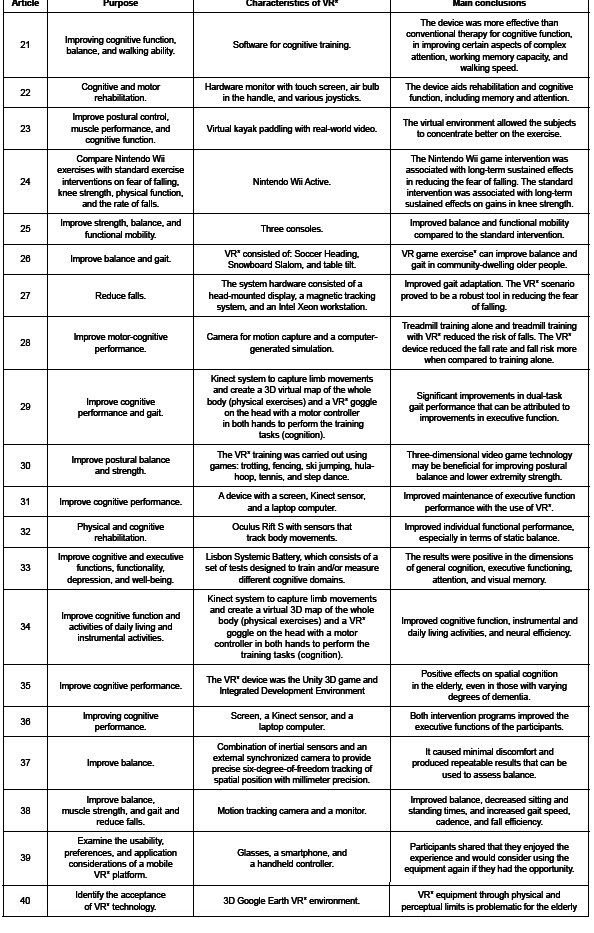
Description of the selected studies (20) in terms of purpose, VR* characteristics, main conclusions, and adverse effects. Fortaleza, CE, Brazil, 2023

 The main purposes, characteristics, and conclusions of the studies are described in Figure 4 . 

*VR = Virtual Reality

*VR = Virtual Reality

The characteristics of the non-immersive devices included the use of screens, computers, mice, videos, joysticks, and Kinect. Each VR session ranged from 40 seconds to 60 minutes, most of which were carried out three times a week, lasting a minimum of six weeks and a maximum of 12. As for the immersive devices, they used VR goggles and sensors, and each session ranged from 30 seconds to 15 minutes, with breaks between sessions to avoid cyber-sickness. Most of them took place three times a week, lasting a minimum of one day and a maximum of three weeks.

 Concerning discomfort when using VR, the majority of the studies (16 articles) ^(^ 21 ^-^ 34 ^,^ 36 ^,^ 38 ^)^ did not report whether or not it was present, which is a limitation when discussing the data, given that the use of this technology by the population can cause invasive effects. Only four studies reported dizziness, nausea, and anxiety as the most prevalent effects ^(^ 35 ^,^ 37 ^,^ 39 ^-^ 40 ^)^ . To minimize these effects, the researchers used specific interventions, such as the use of a modern display head, a padded platform ^(^ 37 ^)^ , and seated interventions until they felt confident to stand up, in addition to professionals at their side to prevent falls ^(^ 39 ^)^ ; the use of a chair to handle VR ^(^ 40 ^)^ . In addition, some studies have pointed out interventions to minimize adverse effects such as an interval of more than one minute between sessions when using the device ^(^ 32 ^)^ , and if participants experienced syncope or fatigue, a chair was provided so they could rest ^(^ 30 ^)^ . It is worth noting that of the studies that reported adverse effects, all were immersive devices ^(^ 35 ^,^ 37 ^,^ 39 ^-^ 40 ^)^ . 

**Figure 5 -:**
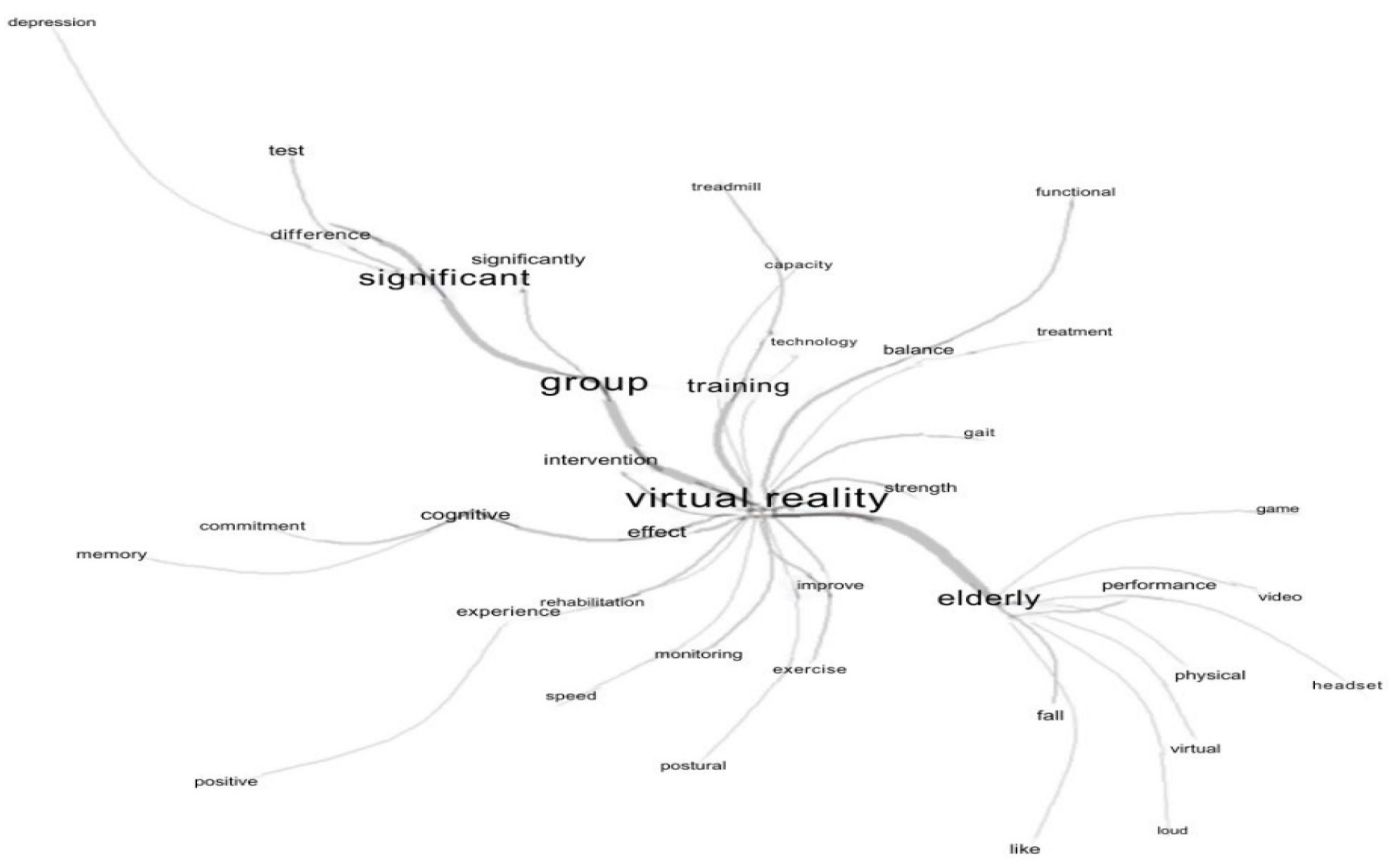
Similarity analysis of the selected articles. Fortaleza, CE, Brazil, 2023

 The analysis of similarity resulted in the generation of a maximum tree which made it possible to identify the interrelationship between the terms “virtual reality” and “elderly”. The analysis revealed that the central term “virtual reality” (n=206) served as an organizing structure, involving ten associated terms: strength (n=19), gait (n=19), balance (n=40), training (n=96), group (n=186), intervention (n=34), cognitive (n=59), effect (n=44), improve (n=31) and with an emphasis on the term elderly (n=192). This, in turn, acted as an intermediate element in the representation, with nine terms linked to it: game (n=17), video (n=31), performance (n=29), headset (n=11), physical (n=19), virtual (n=17), loud (n=11), fall (n=37), like (n=14). The term that also stood out, but more distally, was “group” (n=186), in which four words were related: significant (n=106), difference (n=67), test (n=49) and depression (n=11), as shown in Figure 5

## Discussion

 Mapping the studies made it possible to learn about the purposes and characteristics of VR devices for the elderly. It is noteworthy that the elderly have positive judgments regarding the acceptance and use of VR and identify it as useful, easy to use, and a pleasant experience, which implies beneficial attitudes towards the adoption of VR ^(^ 41 ^)^ . 

 South Korea stands out in the production of studies on VR, which is justified by the high investment in technologies that aim to unite the physical environment with the virtual one, such as the Digital New Deal program created in the country by the Ministry of Science, Information Technology and Communication ^(^ 42 ^)^ . 

 There has been a growing trend in the development of virtual devices in recent years. One study points out that VR technological applications have advanced to a point where they can be applied to various sectors such as education, health, training, and industry, among others, and were initially only used for games or entertainment, which corroborates the results of this research ^(^ 8 ^)^ . 

 Concerning the methodological approaches used, we would highlight the clinical trial, which is considered the gold standard and the highest level of scientific evidence for intervention studies in the health area. Some studies conducted in this way enable the use of decision-making tools by managers, as well as favoring the development of clinical guidelines to help professionals in their practice ^(^ 43 ^)^ . The use of technology by the elderly is expanding and it is essential to develop rigorous, well-conducted studies to better validate the effects of its use. 

 Although this review found only one study involving nurses, it is important to note that there have been advances in research involving the teaching of nursing through VR. Other studies suggest that VR can effectively improve knowledge in nursing education ^(^ 44 ^-^ 45 ^)^ . These results could help in the development of technologies to improve nursing care for the elderly. However, there is still a need to establish partnerships between the areas of technology and health, both in the public and private sectors, as well as support from funding bodies for research and the development of VR solutions for use by nurses in different care settings. 

 In the studies, most of the elderly had preserved cognition and no diseases that could affect the neurological level ^(^ 21 ^-^ 23 ^,^ 26 ^,^ 32 ^,^ 37 ^,^ 39 ^)^ . It is important to highlight the need to develop studies on the use of VR including elderly people with advanced cognitive and/or neurological deficits to provide information on the effectiveness of the technology in this population. This would make it possible to compare the effectiveness of VR in elderly people with preserved cognition or not, and thus generate information that could help professionals during the care they provide. 

 The efficacy of using VR devices with the elderly has been positively observed ^(^ 21 ^-^ 33 ^,^ 35 ^,^ 37 ^)^ . However, the types of devices and interventions vary, making it difficult to compare the studies equally, even though they have been shown to bring about changes in general well-being and quality of life. Recent research has suggested that there are potential benefits of VR interventions for treating pain in various populations, combating social isolation, and improving balance, strength, and cognition ^(^ 14 ^,^ 46 ^-^ 47 ^)^ . 

 As for the VR devices used, the improvement and rehabilitation of cognitive, mobility, and balance functions were found to be prevalent in several studies ^(^ 21 ^-^ 26 ^,^ 28 ^-^ 32 ^,^ 35 ^,^ 37 ^-^ 38 ^)^ . This is in line with the need to improve quality of life, develop independence, and promote health for the elderly in the community, given the changes that occur with aging. In addition, another purpose of using VR with the elderly is entertainment ^(^ 39 ^)^ , participation in leisure activities is also a care tool, as is the case with VR, which allows the elderly to immerse themselves in hiking environments, for example. 

 Regarding the characteristics of the technology, non-immersive VR prevailed in the studies, although this may be related to the costs of carrying out the research, as immersive VR requires a greater investment in both software and hardware requirements. However, despite the benefits of using VR for older people, it is necessary to highlight the invasive effects it causes. As VR becomes more popular, it is necessary to understand its potential repercussions on individuals, both positive and negative ^(^ 48 ^-^ 49 ^)^ . Researchers have pointed out that the use of VR can lead to negative physiological results, such as nausea, dizziness, and eye fatigue ^(^ 50 ^)^ . These effects can result in non-adherence. Special care and attention are needed when developing this type of technology to mitigate the invasive effects on those who use it. 

The study shows that VR technology can be introduced into the daily lives of the elderly, whether in terms of rehabilitation, mental health, cognition, or even entertainment, but it requires expertise and partnerships. It appears to be a promising device for more active aging in an increasingly technological world.

Nurses must therefore keep up with the changes and ensure that they play a leading role in the world of care through knowledge and the use of new technologies. In this sense, the contribution of this study to scientific advancement stands out, as it brings elements that make it possible to visualize and understand the development and use of VR devices as a tool for caring for the elderly.

A limitation is the low number of studies that have reported on the discomfort presented by the use of VR devices, making it difficult to analyze this aspect. However, the highlights of this review are the methodological rigor required by the JBI and the novelty of the research topic.

## Conclusion

South Korea stood out in the development of VR technologies. Nurses were the professionals least involved in the development and use of VR. The main purposes were to improve and/or rehabilitate functions that decline with aging, either physiologically or as a result of illness or disease. In terms of characteristics, there was a greater number of non-immersive devices using screens, computers, mice, videos, joysticks, and Kinect, with sessions ranging from seconds to an hour, held no more than three times a week, lasting up to 12 weeks. As for the immersive devices, they used VR glasses and sensors, with sessions ranging from seconds to 15 minutes, up to three times a week. Finally, it is suggested that further studies be carried out in other environments, such as long-term care facilities for the elderly, senior living centers, and hospitals. In this way, further research could enable a wider use of VR by health professionals, especially nurses, in the care provided to the elderly.
