# Supplementary material for: Purposes and characteristics of virtual reality technologies for the elderly in the community: a scoping review
Source: Rev Lat Am Enfermagem. 2024 Nov 22;32:e4389. doi: 10.1590/1518-8345.7419.4389 (PMC11654048; doi:10.1590/1518-8345.7419.4389)
Supplement: Translation

# Translation: Finalidades y características de las tecnologías de realidad virtual para adultos mayores en la comunidad: revisión de escopo *

**DOI:** 10.1590/1518-8345.7419.4388

**Published:** 2024-11-15

**Authors:** Jamylle Lucas Diniz, Natalia Maria Cavalcante Oliveira, Janaina Fonseca Victor Coutinho, Marília Braga Marques, Carolina Bravo Pillon, Ítalo Linhares de Araújo

**Affiliations:** ^1^ Universidade Federal do Ceará, Departamento de Enfermagem, Fortaleza, CE, Brasil.; ^2^ Becaria de la Coordenação de Aperfeiçoamento de Pessoal de Nível Superior (CAPES), Brasil.; ^3^ Universidade Federal de Pelotas, Centro de Artes, Pelotas, RS, Brasil.; ^4^ Universidade Federal do Ceará, Campus Itapajé, Itapajé, CE, Brasil.

**Keywords:** Tecnología, Realidad Virtual, Anciano, Residencia, Equilibrio Postural, Rehabilitación

## Abstract

mapear las características y las finalidades de las tecnologías de Realidad Virtual (RV) para adultos mayores en la comunidad.

revisión de escopo, según la recomendaciones del JBI ( *Joanna Briggs* *Institute* ) y descrito de acuerdo con el *Preferred Reporting Items for Systematic Reviewas and Meta-Analyses Extension for Scoping Reviews* . Fueron incluidas diez bases de datos y cuatro plataformas referentes a la literatura gris. La selección de los estudios fue realizada después de la remoción de duplicados, evaluación individual y también en pares. El análisis de similitud fue utilizado para identificar las afinidades entre las palabras y sus resultados.

fueron mapeados 20 estudios de RV para los adultos mayores, siendo la mayoría del uso de dispositivos no inmersivos. Las principales finalidades del uso de la RV con adultos mayores fueron mejorar y/o rehabilitar las funciones que entran en declino con el envejecimiento, de forma fisiológica o provenientes de enfermedades o daños. Los dispositivos de RV constituyen una potencial herramienta para la prevención de caídas y disminución cognitiva, y favorecen la ejecución de actividades instrumentales de la vida diaria. El análisis de similitud resultó en la generación del árbol máximo, que permitió identificar la interrelación con los términos “realidad virtual” y “adulto mayor”, como elemento central e intermediario, respectivamente.

se recomienda el desarrollo de nuevos estudios, en otros ambientes, para permitir una mayor amplitud en el uso de la RV, por profesionales de la salud, en especial de los enfermeros, en la asistencia prestada a los adultos mayores.

## Puntos destacados

**(1)** Mejora de la función cognitiva, el equilibrio y la movilidad en personas mayores.

**(2)** Favorece la ejecución de actividades instrumentales de la vida diaria.

**(3)** Se identificó un mayor número de dispositivos no inmersivos.

**(4)** Reconocen el dispositivo RV como útil, fácil de usar y una experiencia agradable.

**(5)** Se recomienda el desarrollo de nuevos estudios en otros entornos sanitarios.

## Introducción

 Formas innovadoras y complementarias para los cuidados en salud están siendo desarrolladas por medio de soluciones tecnológicas dirigidas al auxilio, manutención y rehabilitación de adultos mayores, entre las cuales se destacan: *Internet of Health Things* (IoHT) ^(^ 1 ^)^ , dispositivos usables ( *wearables)* ^(^ 2 ^)^ *,* Realidad Aumentada (RA), Realidad Virtual (RV) y Realidad Mixta (RM) ^(^ 3 ^-^ 6 ^)^ . 

 En lo que se refiere a las tecnologías virtuales nos interesan los dispositivos de RV, porque posibilitan diversificar las experiencias dependiendo del individuo y del ambiente. Los estudios de intervención con uso de la RV ya fueron utilizados en el área de la educación, durante la pandemia de la COVID-19 y en el área de la salud para el tratamiento del dolor crónico, para la rehabilitación después de un Accidente Vascular Cerebral (AVC) y para el control de síntomas de depresión ^(^ 7 ^-^ 10 ^)^ . 

 En 2020, el mercado global de RV utilizó, aproximadamente US$ 10,85 billones; es posible que en 2028 pueda alcanzar US$ 52,03 billones, apuntando un crecimiento de 21,9%. América del Norte se destaca en el desarrollo y en la comercialización, siendo los sectores de la salud y de la educación los que más demandan el desarrollo de dispositivos de RV ^(^ 11 ^)^ . 

 La RV puede ser utilizada por diferentes grupos etarios; sin embargo, se observa una diferencia en relación a la demanda, la población joven la busca para el entretenimiento ^(^ 12 ^)^ , en cuanto para adultos mayores es considerada como una herramienta de cuidado de los profesionales de la salud, especialmente, para mejorar los aspectos: físicos (postura, equilibrio, marcha, amplitud de movimiento y gestos), cognitivos (función executiva, atención, memoria, síntomas de depresión, ansiedad, humor) ^(^ 13 ^)^ y sociales. 

 Los dispositivos de RV muestran potencial para realizar cambios positivos en el bienestar y en la calidad de vida de adultos mayores ^(^ 14 ^-^ 15 ^)^ ; sin embargo, no son libres de riesgo, ya que pueden causar incomodidad durante el uso, como náuseas, mareo, vértigo y dolores de cabeza. Estos síntomas son denominados de “náusea digital”, que pueden estar relacionados al tipo de dispositivo, tiempo de exposición, predisposición y a la no adaptación ^(^ 16 ^)^ . 

 Los dispositivos de RV para adultos mayores presentan diferentes características y finalidades, conocerlas permitirá a los profesionales de la salud, en especial al enfermero, evaluar las nuevas posibilidades de intervenciones y la mejoría del cuidado ofrecido. Así, al investigar y mapear, en la literatura los dispositivos de RV, se pueden evaluar las posibilidades de su uso, los atributos y las posibles incomodidades. Además de ayudar a elegir el mejor tipo, sabiendo cuáles son los más aceptados y adecuados, también proporciona la información para crear listas de verificación estandarizadas, con su propia interfaz y especificaciones de funcionalidad para su desarrollo dirigidos específicamente a la población mayor ^(^ 12 ^)^ . 

De esa forma, el objetivo de este estudio fue mapear las características y las finalidades de las tecnologías de realidad virtual para adultos mayores de la comunidad.

## Método

### Delineamiento del estudio

 Se trata de una revisión de escopo (RE) que adoptó las recomendaciones de la *Joanna Briggs Institute* (JBI) ^(^ 17 ^)^ , registradas en la *Open Science Framework* con DOI 10.17605/OSF.IO/YGZ9Q. Fue seguido el *Reporting Items for Systematic Reviews and Meta-analyses Extension for Scoping Reviews* (PRISMA-ScR) ^(^ 18 ^)^ . 

### Escenario en que se realizó la recolección de datos

 La búsqueda fue realizada en las bases de datos: *Medical Literature Analysis and Retrieval System Online/National Library of Medicine* (MEDLINE/PubMed), *Cumulative Index to Nursing and Allied Health Literature* (CINAHL), Scopus, Embase, JBI *Evidence Synthesis* , Epistemonikos, Compendex, PsycINFO, *Cochrane Library* y *Web of Science* . En la literatura gris las fuentes utilizadas fueron: *Google Scholar* (diez primeras páginas); Continente Europeo – *System for Information on Grey Literature in Europe* ( *OpenGrey* ); *ProQuest Global Dissertations and Theses* (diez primeras páginas) 

### Período

La investigación fue realizada entre los meses de abril y mayo de 2023.

### Población

Se utilizó el nemónico Población; Concepto y Contexto (PCC), Población (P) – adultos mayores caracterizados por individuos con edad igual o mayores de 60 años. Concepto (C), uso de tecnologías de RV y el Contexto (Ct) – comunidad (estudios realizados en ambientes comunitarios, o que abarcan el domicilio, no contemplando adultos mayores bajo cuidados institucionales). La pregunta de la investigación elaborada fue: ¿Cuáles son las características y la finalidad de las tecnologías de realidad virtual desarrolladas para adultos mayores en la comunidad?

### Criterios de selección

 Los criterios de elegibilidad fueron organizados utilizando la estrategia PCC ^(^ 17 ^)^ . Fueron incluidos los estudios que investigaron el uso de tecnologías de RV por adultos mayores de la comunidad, sin restricción en cuanto al año de publicación y al idioma. Fueron excluidos los estudios de revisión, cartas, anales de congresos, protocolos de estudios, investigaciones realizadas en hospitales e Instituciones de Larga Permanencia (por comprender adultos mayores en cuidados institucionales) y artículos duplicados. 

### Definición de la muestra

 Fueron utilizados los descriptores controlados de los vocabularios de los Descriptores en Ciencias de la Salud (DeCS), *Medical Subject Heading* (MeSH) y *Emtree* y palabras clave. Para esto, fue realizada una investigación simple en la MEDLINE/PubMed y en la Scopus, utilizando los descriptores del PCC, con la finalidad de analizar las palabras contenidas en el título y en el resumen y en la indexación de los términos usados para describir los artículos. Esta fase fue realizada por la investigadora con la ayuda de un bibliotecario de la Universidad Federal del Ceará. Posteriormente, fueron creados los comandos de investigación con las palabras recuperadas juntamente con los descriptores, abarcando sus sinónimos y las palabras clave, conforme su relevancia para el estudio. El cruzamiento fue mediado por los operadores booleanos *AND* y *OR* . Así, la elaboración de la estrategia de búsqueda fue estructurada en cinco etapas: extracción, conversión, combinación, construcción y uso ^(^ 19 ^)^ , como presentado en la Figura 1 . 

**Figura 1 -:** Elaboración de la estrategia de búsqueda en las bases de datos y en la literatura gris. Fortaleza, CE, Brasil, 2023

### Recolección de datos

 Los procedimientos de búsqueda, evaluación, selección, caracterización y análisis contaron con dos investigadoras, que de forma pareada y con reuniones para obtener consenso trataban sobre el proceso de la toma de decisiones e inclusión de los artículos. Se utilizó el aplicativo *Rayyan* , desarrollado por el *Qatar Computing Research Institute* (QCRI), para auxiliar en el archivamiento, organización y selección. 

### Variables del estudio

Las variables utilizadas fueron: autor, año de publicación, idioma, país de origen, tipo de estudio, profesionales participantes, características de las tecnologías, finalidad de la RV, efectos colaterales provenientes del uso de la RV y principales conclusiones.

### Instrumentos utilizados para la recolección de informaciones

El mapeo de las informaciones fue establecido con base en el instrumento de la JBI para caracterizar las producciones.

### Tratamiento y análisis de los datos

 Los resultados fueron sometidos al análisis descriptivo utilizado, el *software Interface de R pour les Analyses Multidimensionnelle de Textes et de Questionnaires* (IRAMuTeQ), 0.7 Alpha 2. Se realizó el análisis de similitud, caracterizado por posibilitar la identificación de co-ocorrencias entre las palabras y su resultado, indicando la conectividad entre las mismas por medio de grafos ^(^ 20 ^)^ . 

### Aspectos éticos

Se trata de un estudio que utiliza datos secundarios, de esa forma no fue necesaria la apreciación del Comité de Ética en Investigación (CEI).

## Resultados

 Fueron identificados 4.839 artículos en las bases de datos y 330 en la literatura gris, totalizando 5.169, de los cuales fueron excluidos 2.291 por publicaciones duplicadas, restando 2.878 para la lectura de título y resumen. Después del primer análisis, con la lectura de títulos y resúmenes clasificados, fueron excluidos 2.848 y seleccionados 360 para lectura completa. De estos, 340 fueron excluidos por no responder a la pregunta de la investigación. La muestra final estuvo compuesta de 20 artículos ^(^ 21 ^-^ 40 ^)^ , conforme presentado en la Figura 2 . 

**Figura 2 -:** Diagrama de flujo de la selección de los estudios. Fortaleza, CE, Brasil, 2023

 En cuanto al país de origen de los estudios seis fueron realizados en Corea del Sur ^(^ 21 ^-^ 23 ^,^ 26 ^,^ 30 ^,^ 38 ^)^ , tres en Grecia ^(^ 27 ^,^ 31 ^,^ 36 ^)^ , dos en los EUA ^(^ 37 ^,^ 39 ^)^ y en Taiwán ^(^ 29 ^,^ 34 ^)^ , y uno en Singapur ^(^ 24 ^)^ , Irán ^(^ 25 ^)^ , Polonia ^(^ 32 ^)^ , Portugal ^(^ 33 ^)^ , Canadá ^(^ 35 ^)^ , Australia ^(^ 40 ^)^ , respectivamente, y un estudio multicéntrico ^(^ 28 ^)^ en Bélgica, Israel, Italia, Holanda y Reino Unido. En cuanto al año de publicación, cuatro artículos fueron publicados en 2020 ^(^ 22 ^,^ 33 ^-^ 35 ^)^ y 2017 ^(^ 30 ^-^ 31 ^,^ 37 ^,^ 40 ^)^ , y tres en 2019 ^(^ 23 ^,^ 29 ^,^ 39 ^)^ . Seguidos de dos en 2021 ^(^ 21 ^,^ 25 ^)^ , en 2016 ^(^ 24 ^,^ 28 ^)^ y uno en 2022 ^(^ 32 ^)^ , 2018 ^(^ 36 ^)^ , 2015 ^(^ 26 ^)^ , 2013 ^(^ 38 ^)^ y 2007 ^(^ 27 ^)^ , respectivamente. 

 Los instrumentos utilizados en los estudios fueron el Mini Examen de Estado Mental (MEEM); el Test Muscular Manual de las extremidades superior e inferior; el *Montreal Cognitive Assessment* (MOCa); la Escala de Eficacia de Caídas Modificada (MFES); la Escala de Evaluación Clínica de Demencia y la Escala del Equilibrio de Berg. La descripción de los estudios conforme a la población, criterios de inclusión y profesional participante se presentan condensadas en la Figura 3 . 

 Las principales finalidades, características y conclusiones de los estudios se describen en la Figura 4 . 

**Figura 3 -:** Descripción de los estudios seleccionados (20) de acuerdo con autor, tipo de estudio, población, tipo de RV* y profesional participante. Fortaleza, CE, Brasil, 2023

*RV = Realidad Virtual

**Figura 4 -:** Descripción de los estudios seleccionados (20) en cuanto a la finalidad, características de la RV*, principales conclusiones y efectos adversos. Fortaleza, CE, Brasil, 2023

*RV = Realidad Virtual

 En relación a las características de los dispositivos no inmersivos se destacan el uso de pantallas, computadores, *mouses* , videos y *joysticks* y *Kinect* ; cada sesión de RV variaba de 40 segundos a 60 minutos, la mayoría realizada tres veces por semana, con duración mínima de seis semanas y máxima de 12. Algunas intervenciones eran iniciadas con la presentación/ensayos del dispositivo ^(^ 23 ^,^ 25 ^)^ . En cuanto a los dispositivos inmersivos, estos utilizaban anteojos de RV y sensores; cada sesión variaba de 30 segundos a 15 minutos, con intervalos entre las sesiones para evitar la nausea cibernética. La mayoría fue realizada tres veces por semana, con duración mínima de un día y máxima de tres semanas. 

 En lo que se refiere a la incomodidad en el uso de la RV, la mayoría de los estudios (16 artículos) ^(^ 21 ^-^ 34 ^,^ 36 ^,^ 38 ^)^ no relataron su presencia, lo que presenta una limitación para la discusión de los datos, ya que el uso de esa tecnología por la población puede causar efectos invasivos. Apenas cuatro estudios apuntaron el mareo, la nausea y la ansiedad como los efectos más prevalentes ^(^ 35 ^,^ 37 ^,^ 39 ^-^ 40 ^)^ . Para minimizar esos efectos, los investigadores utilizaron las intervenciones específicas, como: uso de cabezote moderno de exhibición, plataforma acolchada ^(^ 37 ^)^ , intervenciones sentadas hasta que se sintiesen confianza de quedar en pie; también habían profesionales al lado del sujeto para evitar caídas ^(^ 39 ^)^ ; y, uso de sillas para manosear la RV ^(^ 40 ^)^ . Además de eso, algunos estudios apuntaron intervenciones para minimizar efectos adversos como intervalo entre las sesiones mayor que un minuto al usar el dispositivo ^(^ 32 ^)^ ; si los participantes llegasen a experimentar desmayo o fatiga, fue suministrada una silla para que pudiesen descansar ^(^ 30 ^)^ . Vale destacar, que los estudios que apuntaron los efectos adversos, todos eran dispositivos inmersivos ^(^ 35 ^,^ 37 ^,^ 39 ^-^ 40 ^)^ . 

 El análisis de similitud resultó en la generación del árbol máximo que permitió identificar la interrelación entre los términos “realidad virtual” y “adulto mayor”. El análisis reveló que el término central “realidad virtual” (n=206) sirvió como estructura organizadora, contemplando diez términos asociados: fuerza (n=19), marcha (n=19), equilibrio (n=40), entrenamiento (n=96), grupo (n=186), intervención (n=34), cognitivo (n=59), efecto (n=44), mejorar (n=31) y énfasis en el término adulto mayor (n=192). Este, a su vez, actuó como elemento intermediario de la representación, con nueve términos vinculados a él: juego (n=17), video (n=31), desempeño (n=29), audífonos (n=11), físico (n=19), virtual (n=17), alto (n=11), caída (n=37) y gustar (n=14). El término que también se destacó - sin embargo más distal - fue “grupo” (n=186), en el cual fueron relacionadas cuatro palabras: significativo (n=106), diferencia (n=67), test (n=49) y depresión (n=11); como mostrado en la Figura 5 . 

**Figura 5 -:** Análisis de similitud de los artículos seleccionados. Fortaleza, CE, Brasil, 2023

## Discusión

 El mapeo de los estudios posibilitó conocer las finalidades y las características de los dispositivos de RV para adultos mayores. Se destaca que los adultos mayores tienen juzgamientos positivos en relación a la aceptación y utilización de la RV y a identificarla como útil, fácil de usar, experiencia agradable, lo que implica en actitudes benéficas en relación a la adopción de la RV ^(^ 41 ^)^ . 

 Corea del Sur se destaca en la producción de estudios sobre RV, hecho justificado por la alta inversión en tecnologías que objetivan unir el ambiente físico al virtual, como es el caso del programa *Digital New Deal* , creado en ese país por el Ministerio de la Ciencia, Tecnología de la Información y Comunicación ^(^ 42 ^)^ . 

 Se observó una tendencia de crecimiento en el desarrollo de dispositivos virtuales en los últimos años. Un estudio apunta que las aplicaciones tecnológicas de la RV avanzaron a tal punto que estas pueden ser aplicadas en diversos sectores como: educación, salud, entrenamientos e industrias, entre otros, siendo inicialmente utilizadas apenas para juegos o entretenimiento, lo que es corroborado con los resultados de esta investigación ^(^ 8 ^)^ . 

 En relación a los abordajes metodológicos utilizados, se destaca el ensayo clínico, considerado estándar oro y el más alto nivel de evidencia científica para los estudios de intervención en el área de la salud. Algunos estudios así realizados, posibilitan el uso de herramientas para la toma de decisiones por parte de gestores, así como también favorecen el desarrollo de directrices clínicas para auxiliar a los profesionales en su práctica ^(^ 43 ^)^ . El uso de la tecnología por adultos mayores se encuentra en expansión, siendo esencial el desarrollo de estudios rigurosos y bien realizados para validar mejor los efectos de su utilización. 

 A pesar de que esta investigación encontró apenas un estudio envolviendo al profesional enfermero, es importante destacar que existen avances en investigaciones que incluyen la enseñanza de la Enfermería por medio de la RV. Otros estudios sugieren que la RV puede, efectivamente, mejorar el conocimiento de la educación en Enfermería ^(^ 44 ^-^ 45 ^)^ . Esos resultados podrán ayudar a desarrollar tecnologías para calificar la asistencia del enfermero en el cuidado al adulto mayor. Sin embargo, todavía existe la necesidad de establecer asociaciones entre las áreas de tecnología y salud, tanto en el sector público como en el privado, además del apoyo de los órganos de estímulo a la investigación y al desarrollo de soluciones RV para uso por enfermeros en los diferentes escenarios del cuidar. 

 En los estudios, la mayor parte de los adultos mayores presentaban cognición preservada y ausencia de enfermedades que podrían afectar el nivel neurológico ^(^ 21 ^-^ 23 ^,^ 26 ^,^ 32 ^,^ 37 ^,^ 39 ^)^ . Es importante destacar la necesidad de desarrollar estudios sobre el uso de RV incluyendo adultos mayores con déficit cognitivo y/o neurológicos avanzados, con la finalidad de producir informaciones sobre la efectividad de la tecnología en esa población; esto permitiría comparar la eficacia de la RV en adultos mayores con cognición preservada o sin ella; así se podría generar informaciones que ayudaran a los profesionales durante la asistencia prestada. 

 Se observó, de forma positiva, la eficacia del uso de los dispositivos de RV en adultos mayores ^(^ 21 ^-^ 33 ^,^ 35 ^,^ 37 ^)^ . Sin embargo, los tipos de dispositivos y las intervenciones son variables, dificultando la comparación de forma igualitaria de los estudios; a pesar de eso, se mostraron capaces de provocar cambios en el bienestar general y en la calidad de vida. Investigaciones recientes sugieren que existen beneficios potenciales de las intervenciones de RV para el tratamiento del dolor en diversas poblaciones y para combatir el aislamiento social, mejorar el equilibrio, la fuerza y la cognición ^(^ 14 ^,^ 46 ^-^ 47 ^)^ . 

 En cuanto a la finalidad de los dispositivos de RV aplicados, se observó la mejoría y la rehabilitación de las funciones cognitivas, de movilidad y de equilibrio, que tuvieron prevalencia en varios estudios ^(^ 21 ^-^ 26 ^,^ 28 ^-^ 32 ^,^ 35 ^,^ 37 ^-^ 38 ^)^ . Este hecho está en consonancia con la necesidad de mejorar la calidad de vida, desarrollar la independencia y promover la salud en los adultos mayores de la comunidad, teniendo en vista las alteraciones que ocurren con el envejecimiento. Además de eso, otra finalidad del uso de la RV, en adultos mayores, fue el entretenimiento ^(^ 39 ^)^ y la participación en actividades de ocio; también es una herramienta de cuidado, como es el caso de la RV que permitió, por ejemplo, la inmersión de los adultos mayores en ambientes de senderos para caminadas. 

 En relación a las características de la tecnología, la RV no inmersiva prevaleció en los estudios, sin embargo, puede estar relacionado a los costos de ejecución de las investigaciones, ya que la RV inmersiva requiere una mayor inversión, tanto en requisitos de *software* como de *hardware* . Sin embargo, a pesar de los beneficios del uso de la RV por adultos mayores, es necesario evidenciar los efectos invasivos que esta puede causar. A medida que la RV se torna más popular, es necesario comprender sus potenciales repercusiones sobre los individuos, sean estas positivas o negativas ^(^ 48 ^-^ 49 ^)^ . Los investigadores apuntaron que el uso de RV puede implicar en resultados fisiológicos negativos, como náusea, mareo y fatiga ocular ^(^ 50 ^)^ . Esos efectos, pueden resultar en la no adhesión. Es necesario, al desarrollar ese tipo de tecnología, prestar cuidado y atención especial, con la finalidad de mitigar los efectos invasivos en quien la utiliza. 

 En este estudio se percibió que la tecnología RV puede ser introducida en lo cotidiano de los adultos mayores, en lo que se refiere a la rehabilitación, salud mental, cognición y hasta en el entretenimiento; sin embargo esta exige *expertise* y asociaciones. Se lo señala como un dispositivo promisor para un envejecimiento más activo en un mundo cada vez más tecnológico. 

Por lo expuesto, concluimos que el enfermero debe acompañar los cambios y asegurar su protagonismo en el universo del cuidar, por medio del conocimiento y del uso de nuevas tecnologías. En ese sentido, se destaca la contribución de este estudio para el avance científico, ya que contiene elementos que posibilitan visualizar y comprender el desarrollo y uso de dispositivos de RV como herramienta de cuidado al adulto mayor.

Se observa como limitación el bajo cuantitativo de estudios que relataron las incomodidades presentadas por el uso del dispositivo de RV; así, dificultando el análisis sobre ese aspecto. Sin embargo, esta revisión posee como punto de destaque el rigor metodológico requerido por el JBI y lo inédito del tema investigado.

## Conclusión

 Corea del Sur se destacó en el desarrollo de tecnologías de RV. Los enfermeros fueron los profesionales que presentaron menor participación en el desarrollo y uso de la RV. Las principales finalidades fueron mejorar y/o rehabilitar las funciones que disminuyen con el envejecimiento, de forma fisiológica o son provenientes de enfermedades o daños. En cuanto a las características, hubo mayor cuantitativo de dispositivos no inmersivos con el uso de pantallas, computadores, *mouses* , videos, *joysticks* y *Kinect* , sesiones que variaban de segundos a una hora, realizada como, máximo, tres veces por semana, con duración de hasta 12 semanas. En relación a los dispositivos inmersivos, estos utilizaban anteojos de RV y sensores; las sesiones variaban de segundos a 15 minutos, hasta tres veces por semana. Finalmente, se sugiere el desarrollo de nuevos estudios, en otros ambientes, como en Instituciones de Larga Permanencia para Adultos Mayores (ILAM), en centros de convivencia de adultos mayores y en hospitales. Así, nuevas investigaciones podrán permitir una mayor amplitud en el uso de la RV, por profesionales de la salud, en especial por los enfermeros, en la asistencia prestada a los adultos mayores. 

# Translation: Finalidades e características das tecnologias de realidade virtual para os idosos na comunidade: revisão de escopo *

**DOI:** 10.1590/1518-8345.7419.4390

**Published:** 2024-11-15

**Authors:** Jamylle Lucas Diniz, Natalia Maria Cavalcante Oliveira, Janaina Fonseca Victor Coutinho, Marília Braga Marques, Carolina Bravo Pillon, Ítalo Linhares de Araújo

**Affiliations:** ^1^ Universidade Federal do Ceará, Departamento de Enfermagem, Fortaleza, CE, Brasil.; ^2^ Bolsista da Coordenação de Aperfeiçoamento de Pessoal de Nível Superior (CAPES), Brasil.; ^3^ Universidade Federal de Pelotas, Centro de Artes, Pelotas, RS, Brasil.; ^4^ Universidade Federal do Ceará, Campus Itapajé, Itapajé, CE, Brasil.

**Keywords:** Tecnologia, Realidade Virtual, Idoso, Domicílio, Equilíbrio Postural, Reabilitação

## Abstract

mapear as características e as finalidades das tecnologias de Realidade Virtual (RV) para os idosos na comunidade.

revisão de escopo, segundo às recomendações do JBI e descrito de acordo com o *Preferred Reporting Items for Systematic Reviewas and Meta-Analyses Extension for Scoping Reviews* . Foram incluídas dez bases de dados e quatro plataformas referentes à literatura cinzenta. A seleção dos estudos foi realizada após a remoção de duplicatas e a avaliação individual e em pares. A análise de similitude foi utilizada para identificar as concorrências entre as palavras e seus resultados.

foram mapeados 20 estudos de RV para os idosos, sendo a maioria de uso de dispositivos não imersivos. As principais finalidades do uso de RV com os idosos foram melhorar e/ou reabilitar as funções que entram em declínio com o envelhecimento, de forma fisiológica ou advindas de doenças ou agravos. Os dispositivos de RV constituem uma potencial ferramenta para a prevenção de quedas e declínio cognitivo, e favorecem a execução de atividades instrumentais de vida diária. A análise de similitude resultou na geração da árvore máxima, que permitiu identificar a inter-relação com os termos “realidade virtual” e “idoso”, como elemento central e intermediário, respectivamente.

recomenda-se o desenvolvimento de novos estudos, em outros ambientes, o que poderá permitir uma maior amplitude no uso de RV, por profissionais de saúde, em especial dos enfermeiros, na assistência prestada aos idosos.

## Destaques

**(1)** Melhora da função cognitiva, equilíbrio e mobilidade de pessoas idosas.

**(2)** Favorece a execução de atividades instrumentais de vida diária.

**(3)** Identificou-se maior quantitativo de dispositivos não imersivos.

**(4)** Reconhecem o dispositivo RV como útil, fácil de usar e uma experiência agradável.

**(5)** Recomenda-se o desenvolvimento de novos estudos, em outros ambientes de saúde.

## Introdução

 Formas inovadoras e complementares para os cuidados em saúde vêm sendo desenvolvidas por meio de soluções tecnológicas voltadas ao auxílio, à manutenção e à reabilitação de idosos, destacando-se: a *Internet of Health Things* (IoHT) ^(^ 1 ^)^ , dispositivos vestíveis ( *wearables)* ^(^ 2 ^)^ *,* de Realidade Aumentada (RA), de Realidade Virtual (RV) e de Realidade Mista (RM) ^(^ 3 ^-^ 6 ^)^ . 

 No tocante às tecnologias virtuais, interessam-nos os dispositivos de RV que possibilitam diversificar as experiências dependendo do indivíduo e do ambiente. Os estudos de intervenção com uso de RV já foram utilizados na área da educação, durante a pandemia da COVID-19, e na área da saúde para o tratamento de dor crônica, reabilitação após um Acidente Vascular Cerebral (AVC) e controle de sintomas de depressão ^(^ 7 ^-^ 10 ^)^ . 

 Em 2020, o mercado global de RV movimentou aproximadamente US$ 10,85 bilhões e, até 2028 poderá atingir US$ 52,03 bilhões, apontando um crescimento de 21,9%. A América do Norte se destaca no desenvolvimento e na comercialização, sendo os setores da saúde e da educação os que mais demandam o desenvolvimento de dispositivos de RV ^(^ 11 ^)^ . 

 A RV pode ser utilizada por diferentes grupos etários, todavia, observa-se uma diferença em relação à sua demanda: a população jovem busca o entretenimento ^(^ 12 ^)^ , enquanto para os idosos ela é vista como uma ferramenta de cuidado de profissionais de saúde, especialmente na melhoria dos aspectos físicos (postura, equilíbrio, marcha, amplitude de movimento e gastos) cognitivos (função executiva, atenção, memória, sintomas de depressão, ansiedade, humor) ^(^ 13 ^)^ e sociais. 

 Os dispositivos de RV mostram o potencial para as mudanças positivas no bem-estar e na qualidade de vida de idosos ^(^ 14 ^-^ 15 ^)^ , contudo, não são isentos de risco: podem causar desconfortos durante o uso, como náuseas, tontura, vertigem e dores de cabeça. Estes sintomas são denominados de “ciberenjoo”, que pode estar relacionado ao tipo de dispositivo, tempo de exposição, predisposição e não adaptação ^(^ 16 ^)^ . 

 Os dispositivos de RV para os idosos apresentam diferentes características e finalidades e conhecê-las permitirá aos profissionais de saúde, em especial ao enfermeiro, avaliar as novas possibilidades de intervenções e a melhoria do cuidado ofertado. Assim, ao se investigar e mapear na literatura os dispositivos de RV pode-se avaliar as possibilidades de uso, os atributos e os possíveis desconfortos. Além de auxiliar na escolha do melhor tipo, sabendo quais são os mais aceitos e adequados, também fornece informações para criar *checklists* padronizados, com as especificações próprias de interface e funcionalidade para o seu desenvolvimento voltados especificamente para a população idosa ^(^ 12 ^)^ . 

Dessa forma, o objetivo deste estudo foi mapear as características e as finalidades das tecnologias de realidade virtual para os idosos da comunidade.

## Método

### Delineamento do estudo

 Trata-se de uma revisão de escopo (RE) que adotou as recomendações da *Joanna Briggs Institute* (JBI) ^(^ 17 ^)^ , registrada na *Open Science Framework* com DOI 10.17605/OSF.IO/YGZ9Q. Seguiu-se o *Reporting Items for Systematic Reviews and Meta-analyses Extension for Scoping Reviews* (PRISMA-ScR) ^(^ 18 ^)^ . 

### Cenário em que aconteceu a coleta de dados

 A busca foi realizada nas bases de dados *Medical Literature Analysis and Retrieval System Online/National Library of Medicine* (MEDLINE/PubMed), *Cumulative Index to Nursing and Allied Health Literature* (CINAHL), Scopus, Embase, JBI *Evidence Synthesis* , Epistemonikos, Compendex, PsycINFO, *Cochrane Library* e *Web of Science* . Na literatura cinzenta as fontes utilizadas foram: *Google Scholar* (dez primeiras páginas); Continente Europeu – *System for Information on Grey Literature in Europe* (OpenGrey); *ProQuest Global Dissertations and Theses* (dez primeiras páginas) e *ClinicalTrials* . 

### Período

A pesquisa foi realizada entre os meses de abril e maio de 2023.

### População

Utilizou-se o mnemônico População; Conceito e Contexto (PCC), População (P) –idosos caracterizados por indivíduos com idade igual ou maior que 60 anos. Conceito (C), uso de tecnologias de RV e o Contexto (Ct) – comunidade (estudos conduzidos em ambientes comunitários, ou que abranjam o domicílio, não envolvendo idosos sob cuidados institucionais). A questão de pesquisa elaborada foi: Quais as características e a finalidades das tecnologias de realidade virtual desenvolvidas para os idosos na comunidade?

### Critérios de seleção

 Os critérios de elegibilidade foram organizados utilizando a estratégia PCC ^(^ 17 ^)^ . Foram incluídos os estudos que investigaram o uso de tecnologias de RV por idosos da comunidade, sem restrição quanto ao ano de publicação e o idioma. Foram excluídos os estudos de revisão, cartas, anais de congressos, protocolos de estudos, pesquisas conduzidas em hospitais e Instituições de Longa de Permanência, por abranger os idosos em cuidados institucionais, e artigos duplicados. 

### Definição da amostra

 Foram utilizados os descritores controlados dos vocabulários dos Descritores em Ciências da Saúde (DeCS), *Medical Subject Heading* (MeSH) e *Emtree* e palavras-chave. Para isso, foi realizada uma pesquisa simples na MEDLINE/PubMed e na Scopus utilizando os descritores do PCC, com o intuito de analisar as palavras contidas no título e no resumo, além da indexação dos termos usados para descrever os artigos. Esta fase foi realizada pela pesquisadora com a ajuda de um bibliotecário da Universidade Federal do Ceará. Posteriormente, foram criados os comandos de pesquisa com as palavras recuperadas juntamente com os descritores, abrangendo seus sinônimos e as palavras-chave conforme sua relevância para o estudo. O cruzamento foi mediado pelos operadores booleanos *AND* e *OR* . Assim, a elaboração da estratégia de busca foi estruturada em cinco etapas: extração, conversão, combinação, construção e uso ^(^ 19 ^)^ , como apresentado na Figura 1 . 

**Figura 1 -:** Elaboração da estratégia de busca nas bases de dados e na literatura cinzenta. Fortaleza, CE, Brasil, 2023

### Coleta de dados

 Os procedimentos de busca, avaliação, seleção, caracterização e análise contaram com duas pesquisadoras, que de forma pareada e em reuniões para o consenso trataram sobre o processo de tomada de decisão e inclusão dos artigos. Utilizou-se o aplicativo *Rayyan* , desenvolvido pelo *Qatar Computing Research Institute* (QCRI), para auxiliar no arquivamento, na organização e na seleção. 

### Variáveis do estudo

As variáveis utilizadas foram: autoria, ano de publicação, idioma e país de origem, tipo de estudo, profissionais envolvidos, características das tecnologias, finalidade da RV, os efeitos colaterais advindos do uso da RV e as principais conclusões.

### Instrumentos utilizados para a coleta das informações

O mapeamento das informações foi estabelecido com base no instrumento da JBI para caracterizar as produções.

### Tratamento e análise dos dados

 Os resultados foram submetidos à análise descritiva, sendo utilizado o *software Interface de R pour les Analyses Multidimensionnelle de Textes et de Questionnaires* (IRAMuTeQ), 0.7 Alpha 2. Realizou-se a análise de similitude, caracterizada por possibilitar a identificação de coocorrências entre as palavras e seu resultado, trazendo indicações da conexidade entre as mesmas por meio de grafos ^(^ 20 ^)^ . 

### Aspectos éticos

Trata-se de um estudo que utiliza dados secundários, dessa forma não foi necessária a apreciação do Comitê de Ética em Pesquisa (CEP).

## Resultados

 Foram identificados 4.839 artigos nas bases de dados e 330 na literatura cinzenta, totalizando 5.169, dos quais foram excluídos 2.291 por publicações duplicadas, restando 2.878 para a leitura de título e resumo. Após a primeira análise, com a leitura de títulos e resumos triados, foram excluídos 2.848 e selecionados 360 para a leitura na íntegra. Destes, 340 foram excluídos pois não respondiam à pergunta de pesquisa. A amostra final foi composta de 20 artigos ^(^ 21 ^-^ 40 ^)^ , conforme a Figura 2 . 

**Figura 2 -:** Fluxograma de seleção dos estudos. Fortaleza, CE, Brasil, 2023

 Quanto ao país de origem dos estudos, seis foram realizados na Coreia do Sul ^(^ 21 ^-^ 23 ^,^ 26 ^,^ 30 ^,^ 38 ^)^ , três na Grécia ^(^ 27 ^,^ 31 ^,^ 36 ^)^ , dois nos EUA ^(^ 37 ^,^ 39 ^)^ e em Taiwan ^(^ 29 ^,^ 34 ^)^ , e um em Singapura ^(^ 24 ^)^ , Irã ^(^ 25 ^)^ , Polônia ^(^ 32 ^)^ , Portugal ^(^ 33 ^)^ , Canadá ^(^ 35 ^)^ , Austrália ^(^ 40 ^)^ , respectivamente, e um estudo multicêntrico ^(^ 28 ^)^ envolvendo Bélgica, Israel, Itália, Holanda e Reino Unido. Quanto ao ano de publicação, quatro artigos foram publicados em 2020 ^(^ 22 ^,^ 33 ^-^ 35 ^)^ e em 2017 ^(^ 30 ^-^ 31 ^,^ 37 ^,^ 40 ^)^ , e três em 2019 ^(^ 23 ^,^ 29 ^,^ 39 ^)^ , seguidos de dois em 2021 ^(^ 21 ^,^ 25 ^)^ , em 2016 ^(^ 24 ^,^ 28 ^)^ e um em 2022 ^(^ 32 ^)^ , 2018 ^(^ 36 ^)^ , 2015 ^(^ 26 ^)^ , 2013 ^(^ 38 ^)^ e 2007 ^(^ 27 ^)^ , respectivamente. 

 Os instrumentos utilizados nos estudos foram o Miniexame do Estado Mental (MEEM); o Teste Muscular Manual das extremidades superior e inferior; o *Montreal Cognitive Assessment* (MOCa); a Escala de Eficácia de Quedas Modificada (MFES); a Escala de Avaliação Clínica de Demência e a Escala do Equilíbrio de Berg. A descrição dos estudos conforme a população, os critérios de inclusão e o profissional envolvido estão condensados na Figura 3 . 

 As principais finalidades, características e conclusões dos estudos estão descritas na Figura 4 . 

**Figura 3 -:** Descrição dos estudos selecionados (20) de acordo com autor, tipo de estudo, população, tipo de RV* e profissional envolvido. Fortaleza, CE, Brasil, 2023

*RV = Realidade Virtual

**Figura 4 -:** Descrição dos estudos selecionados (20) quanto à finalidade, características da RV*, principais conclusões e efeitos adversos. Fortaleza, CE, Brasil, 2023

*RV = Realidade Virtual.

 Em relação às características dos dispositivos não imersivos, destacam-se o uso de telas, computadores, *mouses* , vídeos, *joysticks* e *Kinect* . Cada sessão de RV variava de 40 segundos a 60 minutos, a maioria realizada três vezes por semana, com a duração mínima de seis semanas e máxima de 12. Algumas intervenções eram iniciadas com a apresentação sobre o dispositivo/aquecimento ^(^ 23 ^,^ 25 ^)^ . Quanto aos dispositivos imersivos, estes utilizavam óculos de RV e sensores. Cada sessão variava de 30 segundos a 15 minutos, com intervalos entre as mesmas para evitar ciberenjoo. A maioria das intervenções foi realizada três vezes por semana, com duração mínima de um dia e máxima de três semanas. 

 No que se refere aos desconfortos com uso da RV, a maioria dos estudos (16 artigos) ^(^ 21 ^-^ 34 ^,^ 36 ^,^ 38 ^)^ não relatou sua presença ou não, o que apresenta uma limitação para a discussão dos dados, visto que o uso dessa tecnologia pela população pode causar efeitos invasivos. Apenas quatro estudos apontaram a tontura, os enjoos e a ansiedade como os efeitos mais prevalentes ^(^ 35 ^,^ 37 ^,^ 39 ^-^ 40 ^)^ . Para minimizar tais efeitos, os pesquisadores utilizaram intervenções específicas, como: uso de cabeçote moderno de exibição, plataforma acolchoada ^(^ 37 ^)^ , intervenções sentadas, até que os participantes se sentissem confiáveis para ficar em pé, além da presença de profissionais ao lado para evitar as quedas ^(^ 39 ^)^ , além de uso da cadeira para manusear a RV ^(^ 40 ^)^ . Além disso, alguns estudos apontaram intervenções para minimizar os efeitos adversos como: intervalo entre as sessões maior que um minuto ao usar o dispositivo ^(^ 32 ^)^ , e oferta de uma cadeira para que os participantes pudessem se sentar caso experimentassem síncope ou fadiga ^(^ 30 ^)^ . Vale destacar que, nos estudos que apontaram os efeitos adversos, todos os dispositivos utilizados eram imersivos ^(^ 35 ^,^ 37 ^,^ 39 ^-^ 40 ^)^ . 

 A análise de similitude resultou na geração da árvore máxima que permitiu identificar a inter-relação entre os termos “realidade virtual” e “idoso”. A análise revelou que o termo central “realidade virtual” (n=206) serviu como estrutura organizadora, envolvendo dez termos associados: força (n=19), marcha (n=19), equilíbrio (n=40), treinamento (n=96), grupo (n=186), intervenção (n=34), cognitivo (n=59), efeito (n=44), melhorar (n=31) e com ênfase no termo idoso (n=192). Este, por sua vez, atuou como elemento intermediário da representação, com nove termos ligados a ele: jogo (n=17), vídeo (n=31), desempenho (n=29), fone de ouvido (n=11), físico (n=19), virtual (n=17), alto (n=11), queda (n=37), gostar (n=14). O termo que se destacou também, porém mais distal, foi “grupo” (n=186), na qual foram relacionadas quatro palavras: significativo (n=106), diferença (n=67), teste (n=49) e depressão (n=11), como mostrado na Figura 5 . 

**Figura 5 -:** Análise de similitude dos artigos selecionados. Fortaleza, CE, Brasil, 2023

## Discussão

 O mapeamento dos estudos possibilitou conhecer as finalidades e as características dos dispositivos de RV para os idosos. Destaca-se que os idosos têm julgamentos positivos em relação à aceitação e à utilização da RV e identificam-na como útil, fácil de usar e uma experiência agradável, o que implica em atitudes benéficas em relação à adoção da RV ^(^ 41 ^)^ . 

 A Coreia do Sul se destaca na produção de estudos sobre RV, fato justificado pelo alto investimento em tecnologias que visam unir o ambiente físico ao virtual, como é o caso do programa *Digital New Deal* , criado no país pelo Ministério da Ciência, Tecnologia da Informação e Comunicação ^(^ 42 ^)^ . 

 Observou-se uma tendência de crescimento no desenvolvimento de dispositivos virtuais nos últimos anos. Um estudo aponta que as aplicações tecnológicas da RV avançaram a um ponto em que podem ser aplicadas aos diversos setores como: educação, saúde, treinamentos, indústrias, entre outros, sendo inicialmente utilizadas apenas para jogos ou entretenimento, o que corrobora os resultados da pesquisa ^(^ 8 ^)^ . 

 Em relação às abordagens metodológicas utilizadas, ressalta-se o ensaio clínico, considerado padrão ouro e o mais alto nível de evidência científica para os estudos de intervenção na área da saúde. Alguns estudos assim conduzidos possibilitam o uso de ferramentas para a tomada de decisões por parte de gestores, bem como favorecem o desenvolvimento de diretrizes clínicas para auxiliar os profissionais na sua prática ^(^ 43 ^)^ . O uso de tecnologia por idosos encontra-se em expansão, sendo essencial o desenvolvimento de estudos rigorosos e bem conduzidos para validar melhor os efeitos da sua utilização. 

 Apesar desta revisão encontrar apenas um estudo envolvendo o profissional enfermeiro, é importante salientar que há avanços em pesquisas que envolvem o ensino da Enfermagem por meio da RV. Outros estudos sugerem que a RV pode efetivamente melhorar o conhecimento na educação em Enfermagem ^(^ 44 ^-^ 45 ^)^ . Tais resultados poderão ajudar no desenvolvimento de tecnologias para qualificar a assistência do enfermeiro ao idoso. No entanto, há ainda a necessidade de se estabelecer parcerias entre as áreas de tecnologia e saúde, tanto no setor público quanto privado, além do apoio dos órgãos de fomento à pesquisa e ao desenvolvimento de soluções em RV para o uso por enfermeiros nos diferentes cenários do cuidado. 

 Nos estudos, a maioria dos idosos apresentava cognição preservada e ausência de doenças que poderiam afetar o nível neurológico ^(^ 21 ^-^ 23 ^,^ 26 ^,^ 32 ^,^ 37 ^,^ 39 ^)^ . É importante salientar a necessidade do desenvolvimento de estudos com o uso de RV incluindo idosos com déficit cognitivo e/ou neurológicos avançados para produzir as informações sobre a efetividade da tecnologia nessa população. Isto permitiria comparar a eficácia da RV em idosos com cognição preservadas ou não, e assim gerar informações que poderão ajudar os profissionais durante a assistência prestada. 

 Observou-se, de forma positiva, a eficácia no uso dos dispositivos de RV com idosos ^(^ 21 ^-^ 33 ^,^ 35 ^,^ 37 ^)^ . No entanto, os tipos de dispositivos e as intervenções são variáveis, dificultando a comparação dos estudos de forma igualitária; apesar disso, mostraram-se capazes de provocar mudanças no bem-estar geral e na qualidade de vida. Pesquisas recentes sugeriram que existem benefícios potenciais das intervenções de RV para o tratamento da dor em diversas populações, para combater o isolamento social e para melhorar o equilíbrio, a força e a cognição ^(^ 14 ^,^ 46 ^-^ 47 ^)^ . 

 Quanto à finalidade dos dispositivos de RV aplicados, observou-se a melhora e a reabilitação das funções, sejam elas cognitivas, de mobilidade, de equilíbrio, e que tiveram prevalência em vários estudos ^(^ 21 ^-^ 26 ^,^ 28 ^-^ 32 ^,^ 35 ^,^ 37 ^-^ 38 ^)^ . Tal fato está em consonância com a necessidade de melhorar a qualidade de vida, desenvolver a independência e promover a saúde dos idosos da comunidade, tendo em vista as alterações que ocorrem com o envelhecimento. Além disso, outra finalidade do uso da RV com idosos foi o entretenimento ^(^ 39 ^)^ : a participação em atividades de lazer também é ferramenta de cuidado, como é o caso da RV, que permitiu, por exemplo, a imersão dos idosos em ambientes de trilha para caminhada. 

 Em relação às características da tecnologia, a RV não imersiva prevaleceu nos estudos, o que, todavia, pode estar relacionado aos custos de execução das pesquisas, pois a RV imersiva requer maior investimento, tanto em requisitos de *software* como de *hardware* . No entanto, apesar dos benefícios do uso da RV por pessoas idosas, é necessário evidenciar os efeitos invasivos que esta causa. À medida que a RV se torna mais popular, é necessário compreender as suas potenciais repercussões sobre os indivíduos, sejam elas positivas e negativas ^(^ 48 ^-^ 49 ^)^ . Os pesquisadores apontaram que o uso de RV pode implicar em resultados fisiológicos negativos, como náusea, tontura e fadiga ocular ^(^ 50 ^)^ . Tais efeitos podem resultar na não adesão. É necessário, ao se desenvolver esse tipo de tecnologia, ter cuidado e atenção especial, com o intuito de mitigar os efeitos invasivos em quem a utiliza. 

 Percebe-se, com o estudo desenvolvido, que a tecnologia RV pode ser introduzida no cotidiano dos idosos, seja no que se refere à reabilitação, à saúde mental, à cognição e até ao entretenimento, porém demanda *expertise* e parcerias. Aponta-se a RV como dispositivo promissor para um envelhecimento mais ativo em um mundo cada vez mais tecnológico. 

Assim, o enfermeiro deve acompanhar as mudanças e assegurar seu protagonismo no universo do cuidar por meio do conhecimento e do uso de novas tecnologias. Nesse sentido, destaca-se a contribuição deste estudo para o avanço científico, pois traz elementos que possibilitam visualizar e compreender o desenvolvimento e o uso de dispositivos de RV como ferramentas de cuidado ao idoso.

Observa-se como limitação o baixo quantitativo de estudos que relataram os desconfortos apresentados pelo uso do dispositivo de RV, dificultando a análise sobre esse aspecto. No entanto, esta revisão possui como ponto de destaque o rigor metodológico requerido pelo JBI e o ineditismo no tema pesquisado.

## Conclusão

 A Coreia do Sul destacou-se no desenvolvimento de tecnologias de RV. Os enfermeiros foram os profissionais que apresentaram menor participação no desenvolvimento e uso de RV. As principais finalidades foram melhorar e/ou reabilitar as funções que entram em declínio com o envelhecimento, de forma fisiológica ou advindas de doenças ou agravos. Quanto às características, houve maior quantitativo de dispositivos não imersivos com o uso de telas, computadores, *mouses* , vídeos, *joysticks* e *Kinect* , sessões que variavam de segundos a uma hora, realizadas no máximo três vezes por semana, com duração de até 12 semanas. Em relação aos dispositivos imersivos, estes utilizavam óculos de RV e sensores, em sessões que variavam de segundos a 15 minutos, realizadas até três vezes por semana. Por fim, sugere-se o desenvolvimento de novos estudos, em outros ambientes, como em Instituições de Longa Permanência para Idosos (ILPI), centros de convivência de idosos e hospitais. Assim, novas pesquisas poderão permitir uma maior amplitude no uso de RV por profissionais de saúde, em especial enfermeiros, na assistência prestada aos idosos.
